# Teaching Case 2-2018: Sclerosing myxopapillary ependymoma mimicking whorling-sclerosing meningioma

**DOI:** 10.5414/NP301099

**Published:** 2018-02-13

**Authors:** Julia Lang, Thomas Czech, Irene Slavc, Dominik Reisinger, Sophie Bartsch, Johannes A. Hainfellner, Christine Haberler, Ellen Gelpi

**Affiliations:** 1 Institute of Neurology,; 2 Department of Neurosurgery,; 3 Department of Pediatrics, and; 4 Department of Biomedical Imaging and Image-guided Therapy, Medical University of Vienna, Vienna, Austria

**Keywords:** myxopapillary ependymoma, whorling-sclerosing meningioma, myxoid, hyaline globules

## Abstract

No Abstract available.

We present unusual neuropathological findings in a 15-year-old male patient. He presented with inguinal pain on the right side for several weeks. MRI revealed an extradural space-occupying lesion at the level of the sacral segments S2 – S4. The lesion showed sparse contrast enhancement and interloped into the right sacral foramen S4/5 (Figure 1). 

During surgical excision the lesion presented as very firm. Histological examination revealed a moderately cellular tumor. The most characteristic feature was the prominence of whorled collagenous and hyalinized structures. These were mostly surrounded by single or small queues of round homogenous tumor cells, but acellular areas consisting of whorls only were also present (Figure 2). These features were reminiscent of whorling-sclerosing variant of meningioma. Most of these whorls appeared to have an even structure, but in some areas they showed a more myxoid appearance. Tumor cells were strongly immunoreactive for GFAP while the hyalinized whorls were spared. The whorls were well depicted with Alcian blue and Gieson stains. EMA immunostaining revealed frequent intracellular lumina and intracytoplasmatic dots characteristic of an ependymal tumor (Figure 2). 

Myxopapillary ependymoma is a variant of ependymoma, which typically occurs in the region of the conus medullaris, cauda equina, and filum terminale [8]. It consists of cuboidal tumor cells lying in characteristic myxomatous or mucoid stroma [1]. The stroma fills the space between tumor cells and delicate blood vessels, and together they are mostly organized around papillary stalks [1, 2] and alternate with more compact regions [3]. A mucoid component surrounds tumor cells and sometimes accumulates within microcysts [4]. While some cases may mimic schwannomas [4], the predominance of fibrous whorls in our case led to the consideration of a whorling-sclerosing variant of meningioma. This meningioma variant is characterized by high amounts of non-calcifying collagen whorls of variable size that may be surrounded by scattered tumor cells [5, 6, 7]. Smear preparations may show solid hyaline masses in a loose background next to areas of tumor cell nests with a whorling appearance [6]. In myxopapillary ependymomas, smears may also contain well-delineated hyaline globules, but they are usually surrounded by tumor cells [2]. 

Extensive tissue sampling to identify areas with typical features of ependymoma and immunohistochemical patterns is helpful to characterize tumor variants with extensive whorling. 

**Figure 1.**
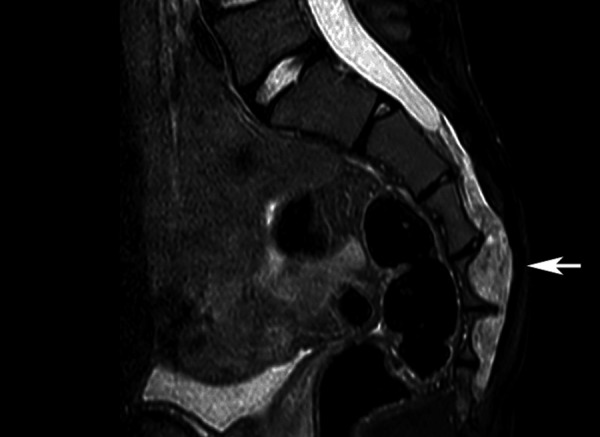
T2-weighted MRI of the extensive extradural tumor at the sacral level.

**Figure 2.**
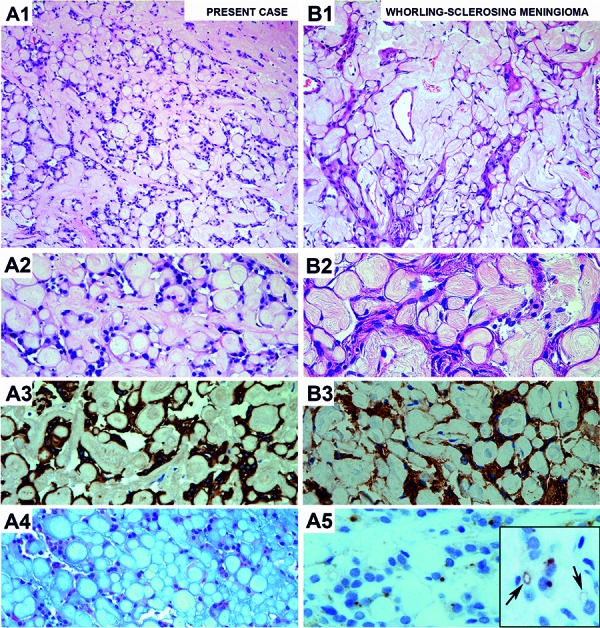
A1, A2, B1, B2: Comparative H & E staining of the present case (A1) and a whorling-sclerosing variant of meningioma (B1) showing similar structures at low magnification. At higher magnification (A2, B2), prominent hyalinized whorling structures are seen in whorling-sclerosing variant of meningioma and the present case. The tumor cells show round nuclei with homogeneous chromatin structure and scarce cytoplasm in all cases. A3: Immunhistochemistry for GFAP shows strong staining of tumor cells while the collagenous whorling structures are spared, indicating the glial nature of this sclerotic tumor type. B3, A5: EMA immunohistochemistry reveals small intracytoplasmic luminae and intracytoplasmatic dots in the present case (A5 and inset, arrows), confirming the ependymal nature of the tumor, while a strong cytoplasmic immunoreactivity of tumor cells are observed in whorling-sclerosing variant of meningioma (B3). A4: Alcian blue staining with strong blue colouring of hyalinized and myxomatous structures in the present case.
